# *Panax ginseng* C.A. Mey. as Multi-Target Regulators of Vascular Homeostasis: Pharmacological Mechanisms and Therapeutic Perspectives

**DOI:** 10.3390/nu18162729

**Published:** 2026-08-20

**Authors:** Yudan Wang, Haiyan Hao, Rui Tan, Zhe Shi, Qinhui Tuo

**Affiliations:** 1 Key Laboratory for Quality Evaluation of Bulk Herbs of Hunan Province, Hunan University of Chinese Medicine, Changsha 410208, China; 20252152@stu.hnucm.edu.cn (Y.W.); haohaiyan0227@163.com (H.H.); m19021495260@163.com (R.T.); 2 Hunan Provincial Key Laboratory of Vascular Biology and Translational Medicine, Changsha 410208, China; 3 Key Laboratory for Novel Antibody Drugs and Intelligent Delivery System of Hunan Province, Hunan University of Medicine, Huaihua 418000, China

**Keywords:** ginseng, ginsenosides, vascular homeostasis, cardiovascular and cerebrovascular diseases

## Abstract

Cardiovascular and cerebrovascular diseases (CVDs) are closely associated with disturbances in vascular homeostasis, including endothelial dysfunction, abnormal vascular remodeling, inflammation, oxidative stress, thrombosis, barrier disruption, and dysregulated angiogenesis. Ginsenosides, the major bioactive saponins of *Panax ginseng* C.A. Mey., have been reported to modulate several of these processes. This review integrates structural, pharmacokinetic, bioinformatic, and experimental evidence to evaluate the vascular actions of representative ginsenosides. Preclinical studies indicate partially overlapping effects on endothelial integrity, vascular tone, permeability, inflammatory and oxidative responses, hemostasis, remodeling, and angiogenesis. Structural differences and biotransformation may contribute to variation in exposure and pharmacological profiles. However, most evidence derives from isolated compounds tested in heterogeneous cell and animal models, direct comparisons or combination studies are limited, and several in vitro concentrations exceed plasma levels reported after oral ginseng administration. The proposed multi-target network should therefore be regarded as hypothesis-generating rather than evidence of coordinated restoration of vascular homeostasis. Future studies should emphasize exposure-relevant experimental designs, comparative assessment of individual ginsenosides and defined combinations, and pharmacokinetic–target engagement relationships.

## 1. Introduction

Cardiovascular and cerebrovascular diseases (CVDs) remain major causes of morbidity and mortality worldwide, accounting for nearly 20 million deaths annually according to the 2026 American Heart Association report [1]. Vascular dysfunction contributes to atherosclerosis, hypertension, stroke, and metabolic vascular complications [2,3]. Vascular homeostasis depends on coordinated interactions among endothelial cells, vascular smooth muscle cells (VSMCs), extracellular matrix components, circulating immune cells, platelets, and coagulation factors, which collectively regulate vascular tone, permeability, hemostasis, remodeling, and angiogenesis [4,5,6]. Disruption of these interactions promotes endothelial dysfunction, inflammation, oxidative stress, thrombosis, barrier leakage, and pathological vascular remodeling. This framework therefore provides a useful basis for evaluating pharmacological agents that affect multiple vascular processes without assuming that modulation of individual pathways is sufficient to restore vascular function.

*Panax ginseng* C.A. Mey. has long been used in East Asian medicine and has been extensively investigated for cardiovascular effects [7]. Ginsenosides are its major bioactive saponins, and individual compounds have been associated with anti-inflammatory, antioxidant, antithrombotic, endothelial-protective, and context-dependent angiogenic effects [8,9,10]. However, the evidence remains predominantly preclinical and heterogeneous. Most studies examine single ginsenosides under different experimental conditions, whereas direct comparisons among compounds, clinically relevant target engagement, and experimental demonstration of additivity or synergy are limited. These limitations complicate interpretation of whether apparently shared effects reflect common pathway convergence, compound-specific actions, or differences among experimental models.

This review integrates structural, pharmacokinetic, bioinformatic, and experimental evidence on ginsenosides within a vascular-homeostasis framework. Particular attention is given to endothelial integrity and remodeling, vascular tone, barrier permeability, platelet and coagulation responses, inflammation and oxidative stress, and angiogenesis. Because metabolic dysfunction intersects with these vascular processes, insulin resistance is also considered as a contributor to vascular injury, together with the emerging neurovascular implications of brain insulin resistance and the proposed concept of “type 3 diabetes” in Alzheimer’s disease [11,12].

## 2. Structural Diversity of Ginsenosides

### 2.1. Structural Classification and Biotransformation

Ginsenosides are characteristic triterpenoid saponins of *Panax ginseng* C.A. Mey. and are classified mainly as dammarane- and oleanane-type compounds. Dammarane ginsenosides are further divided into protopanaxadiol (PPD) and protopanaxatriol (PPT) types. PPD-type compounds, including Rb1, Rb2, Rc, and Rd, are glycosylated mainly at C-3 and C-20, whereas PPT-type compounds, such as Rg1, Re, Rf, and F1, contain an additional hydroxyl group at C-6 and are glycosylated mainly at C-6 and/or C-20. Ro represents the oleanane-type ginsenosides discussed in this review (Table 1) [13].

Processing, enzymatic reactions, and intestinal microbial metabolism generate less glycosylated products such as Rg3, Rh2, Rg5, F1, and compound K (CK), thereby altering polarity, membrane permeability, and metabolic disposition [14,15,16]. These structural differences may contribute to pharmacokinetic and functional variation, but they do not define fixed pharmacological categories because vascular effects overlap across ginsenoside subclasses and depend on dose, exposure, and experimental context.

### 2.2. Disposition and Exposure Limitations

Glycosylation influences the intestinal absorption and disposition of ginsenosides. Highly glycosylated parent compounds generally exhibit limited intestinal permeability, whereas deglycosylation may increase membrane permeability. Systemic exposure is nevertheless determined by multiple factors, including solubility, transporter-mediated efflux, presystemic metabolism, biliary disposition, and microbial biotransformation [17,18,19,20,21,22].

Human pharmacokinetic studies indicate that circulating concentrations after oral administration are generally low. Following Korean red ginseng extract, the reported mean maximum plasma concentrations of Rb1 and CK were 3.94 ± 1.97 and 8.35 ± 3.19 ng/mL, respectively [18]. After administration of 10 g American ginseng root powder, Rb1 reached a peak plasma concentration of 19.90 ± 5.43 ng/mL [17]. Repeated-dose studies likewise detected parent ginsenosides and deglycosylated metabolites predominantly within the ng/mL range, with substantial interindividual variation [19,20].

These exposure data are important when interpreting vascular cell studies. For example, Rb1 at 80 μM and Rb2 at concentrations up to 50 μM, respectively, substantially exceed plasma concentrations observed after oral ginseng administration. Such studies may identify concentration-dependent mechanisms [23,24] but do not establish target engagement at physiologically achievable exposure. Experimental designs should therefore incorporate concentrations informed by human pharmacokinetic data.

### 2.3. Bioinformatics Enrichment and Network Analysis

An exploratory network pharmacology analysis was conducted for 13 representative ginsenosides: Rb1, Rb2, Rc, Rd, Rg1, Re, Rg3, Rh2, CK, F1, Rg5, Ro, and Rk3. Canonical structures and SMILES strings were obtained from PubChem on 30 June 2026. Oral bioavailability and drug-likeness values from TCMSP were recorded descriptively and were not used to exclude compounds.

Potential human targets were retrieved from SwissTargetPrediction and TCMSP on 30 June 2026. SwissTargetPrediction was restricted to Homo sapiens, and predictions with probability > 0.1 were retained. Protein identifiers were mapped to reviewed human UniProt entries and standardized to official gene symbols. For each compound, SwissTargetPrediction and TCMSP targets were combined by union, and the 13 compound-specific target sets were subsequently merged by union. Supplementary Table S1 contains the complete compound-level target records for all 13 ginsenosides included in the analysis.

Vascular-homeostasis-related genes were retrieved from GeneCards and OMIM using the terms “vascular homeostasis”, “endothelial dysfunction”, “vascular inflammation”, “angiogenesis”, and “thrombosis”. GeneCards entries with relevance scores ≥ 10 and genes explicitly associated with relevant OMIM records were retained. Results were combined by union within and between databases and deduplicated by official gene symbol (Supplementary Table S2). Candidate targets were defined as the intersection between the pooled ginsenoside-associated targets and vascular-homeostasis-related genes; no pathway-derived genes were added retrospectively. This procedure yielded 38 intersecting targets, all of which are listed in Supplementary Table S3.

**Figure 1:**
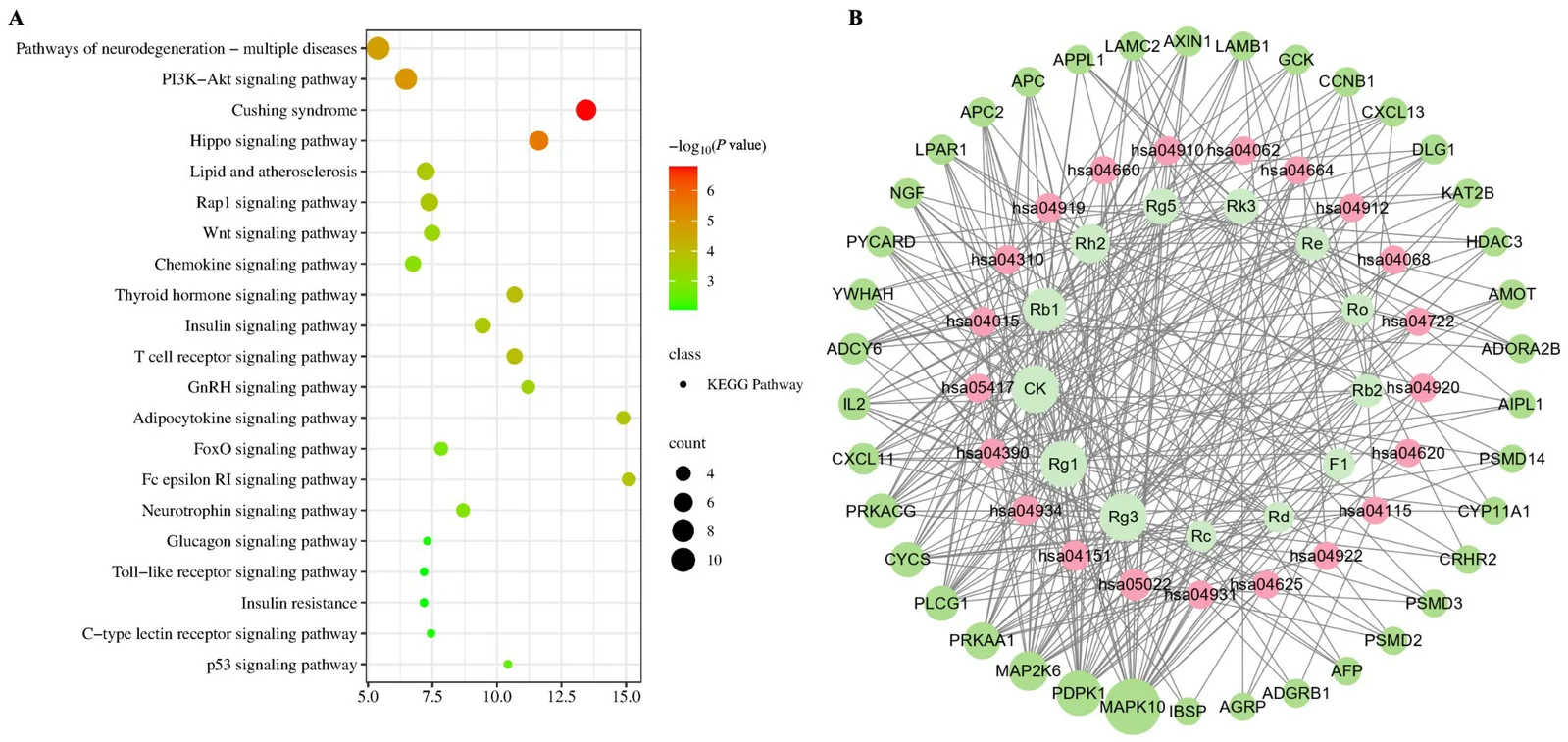
Bioinformatics analysis of representative ginsenosides and vascular-homeostasis-related targets. (**A**) KEGG enrichment of intersecting ginsenoside-associated and vascular-homeostasis-related targets. Dot size represents the number of mapped targets, and color represents the Benjamini–Hochberg-adjusted *p*-value. (**B**) Compound–target–pathway network integrating 13 ginsenosides, intersecting targets, and significantly enriched KEGG pathways. Compound–target edges were derived from SwissTargetPrediction/TCMSP and target–pathway edges from KEGG. Node size is proportional to degree, which represents network connectivity rather than binding affinity, potency, or experimental validation.

KEGG enrichment was performed using clusterProfiler 4.20.0 in R 4.6.1 with KEGG PATHWAY data accessed on 30 June 2026. Gene symbols were converted to Entrez identifiers using org.Hs.eg.db 3.23.1. Homo sapiens was specified as the organism, all KEGG-annotated human genes were used as the background, and pathways containing 10–500 genes were included. Enrichment was assessed by the hypergeometric test with Benjamini–Hochberg correction; adjusted *p* < 0.05 and *q* < 0.05 were considered significant. A total of 21 KEGG pathways met the predefined criteria and were retained for the final analysis. Complete enrichment statistics for these 21 pathways are provided in Supplementary Table S4 and are visualized in Figure 1A.

A compound–target–pathway network was constructed in Cytoscape 3.10.3 using only database-derived compound–target associations and KEGG-derived target–pathway relationships. Literature-inferred or pathway-back-inferred compound–target edges were excluded. Degree, betweenness centrality, and closeness centrality were calculated using NetworkAnalyzer (4.5.0).

Accordingly, the final analytical dataset comprised 13 ginsenosides, 38 intersecting targets, and 21 significantly enriched KEGG pathways, corresponding directly to the compound-level records in Supplementary Table S1, the intersecting-target list in Supplementary Table S3, and the enrichment results in Supplementary Table S4, respectively. MAPK10 showed the highest target degree (26), followed by PDPK1 (22), MAP2K6 (18), PRKAA1 (15), PLCG1 (15), PRKACG (13), and CYCS (13). Among the ginsenosides, Rg3 (degree = 23), CK (22), Rg1 (22), Rb1 (20), and Rh2 (18) showed relatively high connectivity. Degree reflects network topology rather than binding affinity, pharmacological potency, or experimental target validation.

The analysis identifies shared and distinct predicted target–pathway coverage among the selected ginsenosides and is used here as a framework for interpreting the experimental evidence discussed below. It does not establish direct target engagement, functional complementarity, pharmacological synergy, or restoration of vascular homeostasis.

## 3. Disruption of Vascular Homeostasis

Vascular homeostasis is maintained through coordinated interactions among endothelial cells, vascular smooth muscle cells (VSMCs), extracellular matrix components, immune cells, platelets, and coagulation factors, which regulate vascular tone, permeability, hemostasis, remodeling, and angiogenesis [4,5,6]. Endothelial dysfunction is a central feature of this imbalance. Reduced nitric oxide bioavailability impairs vasodilation and favors VSMC contraction and proliferation, whereas disruption of endothelial junctions increases permeability and endothelial activation promotes leukocyte adhesion and a prothrombotic phenotype.

Inflammation, oxidative stress, metabolic abnormalities, and disturbed hemodynamic forces further amplify vascular injury. Reactive oxygen species reduce nitric oxide availability and activate inflammatory signaling, while cytokines and activated immune cells promote additional oxidative stress [22,25]. Platelet and coagulation activation can further reinforce endothelial injury and local inflammation [26,27]. These interacting processes contribute to barrier dysfunction, thrombosis, VSMC remodeling, and abnormal angiogenesis rather than operating as independent linear pathways.

**Figure 2:**
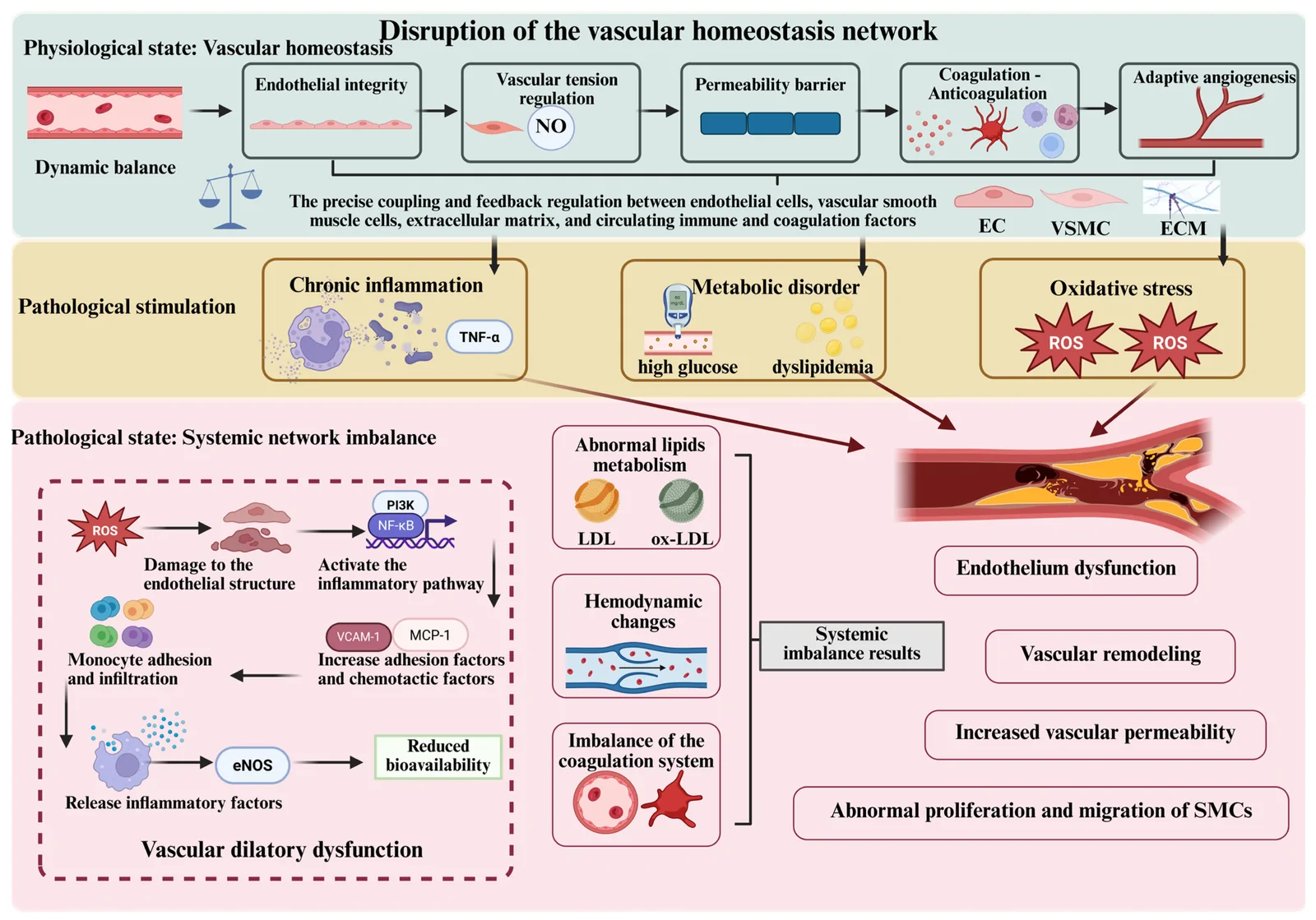
Interconnected pathological domains of vascular homeostasis disruption. EC, endothelial cell; VSMC, vascular smooth muscle cell; ECM, extracellular matrix; ROS, reactive oxygen species; TNF-α, tumor necrosis factor alpha; IL-1β, interleukin-1 beta; IL-6, interleukin-6; LDL, low-density lipoprotein; ox-LDL, oxidized low-density lipoprotein; eNOS, endothelial nitric oxide synthase; NO, nitric oxide; VCAM-1, vascular cell adhesion molecule 1; ICAM-1, intercellular adhesion molecule 1.

The relative importance of these mechanisms varies among vascular disorders. Endothelial dysfunction and lipid-associated inflammation are prominent in atherosclerosis, altered vasomotor control and VSMC remodeling contribute to hypertension, barrier disruption is particularly relevant to stroke and diabetic microvascular disease, and platelet/coagulation activation is central to acute thrombotic events. Figure 2 and Table 2 summarizes these interconnected domains and provides the framework for Section 4, Section 5, Section 6 and Section 7. Because most experimental studies evaluate individual ginsenosides in specific models and assess only selected vascular outcomes, the following sections describe modulation of particular components of vascular homeostasis rather than coordinated restoration of the vascular system as a whole.

## 4. Ginsenoside-Associated Regulation of Vascular Homeostasis

### 4.1. Vascular Structure and Remodeling

Maintenance of vascular structure depends on endothelial viability, controlled vascular smooth muscle cell (VSMC) remodeling, and extracellular matrix homeostasis. Oxidative and endoplasmic reticulum stress, dysregulated apoptosis and autophagy, and VSMC phenotypic switching contribute to vascular injury and remodeling [64]. Relevant experimental findings are summarized in Table 3.

Rb1 has been studied most extensively in this context. Experimental studies associate Rb1 with improved endothelial survival, reduced inflammatory adhesion, modulation of endoplasmic reticulum stress, and enhanced SIRT1/Beclin-1-associated autophagy [21,24,28,65,66,67]. Rg3 has been linked to reduced endothelial activation and inhibition of VSMC proliferation and migration through PPARγ-associated signaling in experimental atherosclerosis [30,32], whereas Rg2 reduced TGF-β1/Smad-dependent fibrosis and ERK1/2-associated vascular remodeling [31,68]. Rd and F1 have also been associated with post-ischemic recovery and endothelial protection, respectively [29,69].

These findings indicate overlap in endothelial stress responses, inflammatory signaling, autophagy, and VSMC remodeling, but the relative emphasis differs among compounds. Because the studies used different disease models, doses, and endpoints, these differences cannot be interpreted as evidence of complementary or synergistic vascular actions.

### 4.2. Regulation of Vascular Tone

Vascular tone reflects the balance between endothelial vasoactive signaling and VSMC contractility. Reduced nitric oxide availability, altered Ca^2+^ handling, neurohumoral activation, and oxidative or inflammatory stress can promote vasoconstriction and vascular remodeling. Experimental evidence is summarized in Table 4.

Rb1 has been associated with increased SIRT1/eNOS signaling and nitric oxide production in endothelial cells and with attenuation of angiotensin II- and TGF-β-related remodeling in vivo [33,70]. However, the 80 μM Rb1 concentration used in endothelial experiments substantially exceeds plasma concentrations reported after oral ginseng administration and should be interpreted as mechanistic rather than exposure-matched evidence.

Ca^2+^ regulation represents another recurrent mechanism. Rd reduced receptor-operated and store-operated Ca^2+^ influx and vascular remodeling [35], while earlier studies also reported modulation of voltage-dependent Ca^2+^ channels by selected ginsenosides [71]. Rg1 attenuated hypoxia-associated pulmonary vasoconstriction and remodeling through pathways involving NF-κB/TGF-β, calpain-1/STAT3, and p38 MAPK [36,37,38]. Additional ischemia-related studies implicate redox, autophagic, and PI3K/Akt-associated mechanisms [44,72,73,74,75,76,77]. At the pathway–family level, these experimental observations correspond to several highly connected nodes in the compound–target–pathway network shown in Figure 1B, including PDPK1 within PI3K/Akt-associated signaling, PLCG1 within phospholipase C/Ca^2+^ signaling, and MAPK10 and MAP2K6 within MAPK-related signaling. This correspondence indicates convergence between the network analysis and experimentally implicated pathway families but does not demonstrate that these individual proteins are directly bound or functionally engaged by the ginsenosides examined in the cited studies.

Overall, the available evidence supports three recurrent levels of regulation—endothelial eNOS/NO signaling, VSMC Ca^2+^ handling, and inflammatory or neurohumoral signaling. These mechanisms have largely been examined in separate models and therefore do not establish a unified vasoregulatory mechanism shared by individual ginsenosides.

**Table 4:** Experimental evidence for ginsenoside-associated regulation of vascular tone.

Type	Model	Biological Indicators	Study Type	Administered Dose	Ref.
Rb1	Replicative-senescent HUVECs	SIRT1, eNOS and NO ↑; caveolin-1 ↓	In vitro	80 μM	[33]
	SD rats with heart failure	Cardiomyocyte cross-sectional area, ANF, β-MHC, collagen I, Ang II, ACE and AT1 ↓; p-Akt/Akt ↑; TGF-β1 and Smad2/3 signaling ↓	In vivo	35/70 mg/kg	[70]
Rd	Stroke-prone renovascular hypertensive rats; basilar artery VSMCs	ROCC- and SOCC-mediated Ca^2+^ influx ↓; basilar arterial remodeling ↓	In vivo/in vitro	20 mg/kg/day	[35]
	MCAO/R model	ROS, MDA, ACSL4, COX-2 ↓; GSH, GPX4, xCT ↑; ZO-1, occludin, claudin-5 ↑; NRG1, ErbB4, p-PI3K, p-Akt, p-mTOR ↑	In vivo	15/30/60 mg/kg/day	[44]
	Spinal cord ischemia–reperfusion rats	Caspase-3, ASK1, JNK ↓	In vivo	6.25/12.5/25/50 mg/kg, i.p.	[75]
Rg3	C57BL/6 mice with hypoxia-induced pulmonary edema	GPX4, Nrf2, HO-1, SLC7A11, FTH1 ↑; TFRC, COX-2, VEGF, HIF-1α, IL-1β, IL-6, TNF-α ↓	In vivo	15/30 mg/kg	[76]
	MCAO/R model; PC12 cells	ROS, MDA ↓; ATP, SOD and HO-1 ↑; Drp1 and Fis1 ↓; Opa1, Mfn1 and Mfn2 ↑	In vivo/in vitro	10/20 mg/kg; 25–125 μM	[72]
Rg1	HPH SD rats; HPAECs	CCN1 ↑; p-NF-κB p65, TGF-β1, p-Smad2/3, TNF-α, IL-1β ↓	In vivo/in vitro	10/20 mg/kg/day; 20 μM	[36]
	HPH C57BL/6 mice; PASMCs	Calpain-1, p-STAT3, IL-6 ↓; mPAP and RVSP ↓	In vivo/in vitro	10/20 mg/kg/day; 0–80 μM	[37]
	Isolated pulmonary arterial rings and PASMCs from SD rats	p38 activation ↓; hypoxia/hypercapnia-induced vasoconstriction ↓	Ex vivo/in vitro	8/40/100 mg/L in arterial-ring experiments	[38]
	MCAO/R model	p62, p-Akt (Ser473), p-rpS6 (Ser240/244), p-ULK1 (Ser757), p-P70S6K ↑; Beclin-1, LAMP1 ↓	In vivo	30 mg/kg/day	[73]
	MCAO/R model	ROS, NOX2, p-PLC, CN, NFAT1, NLRP1 ↓; MAP2 and PSD95 ↑	In vivo	5/10 mg/kg/day	[74]
CK	MCAO/R rats; neuroscreen-1 cells	PTP1B activity ↓; IRS1 tyrosine phosphorylation and PI3K/Akt-associated signaling ↑; neuronal apoptosis ↓	In vivo/in vitro	10 mg/kg; 40 μM	[77]

Note: ↑ and ↓ indicate increases and decreases, respectively, relative to the corresponding model/control comparison reported in the cited study. p-, phosphorylated; ANF, atrial natriuretic factor; β-MHC, beta-myosin heavy chain; Ang II, angiotensin II; ACE, angiotensin-converting enzyme; AT1, angiotensin II type 1 receptor; Akt, protein kinase B; TGF-β1, transforming growth factor beta 1; ROCC, receptor-operated calcium channel; SOCC, store-operated calcium channel; ROS, reactive oxygen species; MDA, malondialdehyde; ACSL4, acyl-CoA synthetase long-chain family member 4; COX-2, cyclooxygenase-2; GSH, glutathione; GPX4, glutathione peroxidase 4; xCT, cystine/glutamate antiporter; ZO-1, zonula occludens-1; NRG1, neuregulin 1; ErbB4, Erb-B2 receptor tyrosine kinase 4; PI3K, phosphoinositide 3-kinase; mTOR, mechanistic target of rapamycin; ASK1, apoptosis signal-regulating kinase 1; JNK, c-Jun N-terminal kinase; Nrf2, nuclear factor erythroid 2-related factor 2; HO-1, heme oxygenase-1; SLC7A11, solute carrier family 7 member 11; FTH1, ferritin heavy chain 1; TFRC, transferrin receptor; VEGF, vascular endothelial growth factor; HIF-1α, hypoxia-inducible factor 1 alpha; IL, interleukin; TNF-α, tumor necrosis factor alpha; ATP, adenosine triphosphate; SOD, superoxide dismutase; Drp1, dynamin-related protein 1; Fis1, mitochondrial fission 1; Opa1, optic atrophy 1; Mfn1/2, mitofusin 1/2; CCN1, cellular communication network factor 1; NF-κB, nuclear factor kappa B; STAT3, signal transducer and activator of transcription 3; mPAP, mean pulmonary arterial pressure; RVSP, right ventricular systolic pressure; rpS6, ribosomal protein S6; ULK1, Unc-51-like kinase 1; P70S6K, p70 ribosomal S6 kinase; LAMP1, lysosome-associated membrane protein 1; NOX2, NADPH oxidase 2; PLC, phospholipase C; CN, calcineurin; NFAT1, nuclear factor of activated T cells 1; NLRP1, NLR family pyrin domain-containing 1; MAP2, microtubule-associated protein 2; PSD95, postsynaptic density protein 95; PTP1B, protein tyrosine phosphatase 1B; IRS1, insulin receptor substrate 1; HPAECs, human pulmonary artery endothelial cells; PASMCs, pulmonary artery smooth muscle cells; HPH, hypoxia-induced pulmonary hypertension; MCAO/R, middle cerebral artery occlusion/reperfusion.

### 4.3. Preservation of Endothelial Barrier Integrity

**Figure 3:**
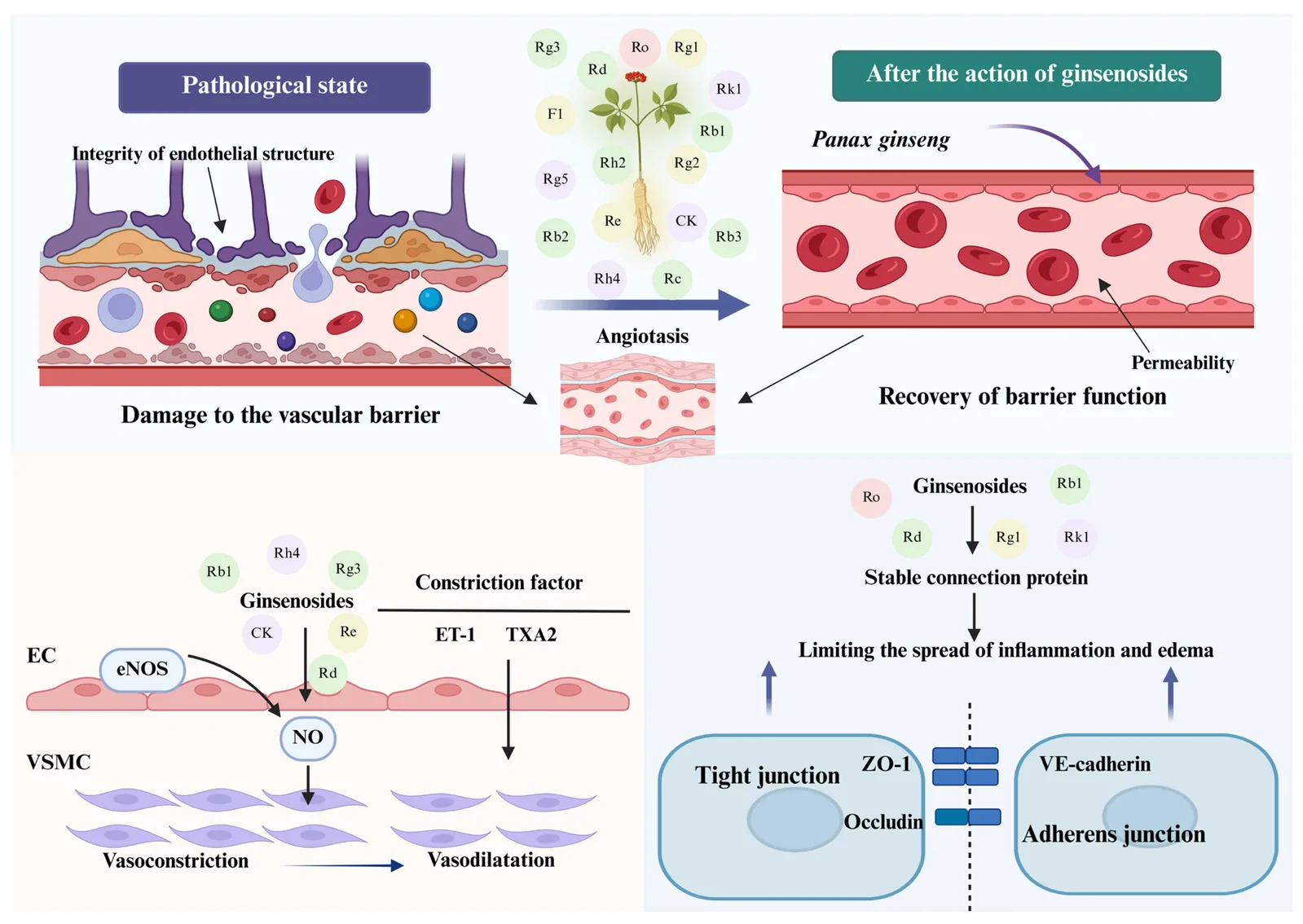
Ginsenoside-associated regulation of endothelial barrier integrity. ET-1, endothelin-1; TXA2, thromboxane A2; EC, endothelial cell; ZO-1, zonula occludens-1; CK, compound K; VEGF, vascular endothelial growth factor; ROS, reactive oxygen species; AMPK, AMP-activated protein kinase; PPARγ, peroxisome proliferator-activated receptor gamma; NF-κB, nuclear factor kappa B.

Endothelial barrier integrity is maintained by tight and adherens junctions, cytoskeletal organization, and regulated transcellular transport. Because barrier properties differ among vascular beds, findings from the blood–brain barrier, retinal microvasculature, intestinal microcirculation, and systemic endothelium should be interpreted within their specific experimental contexts. Relevant studies are summarized in Figure 3 and Table 5.

Rb1 has been associated with preservation of junctional proteins and reduced vascular leakage in cerebral ischemia–reperfusion and mesenteric hyperpermeability models [40,41]. Additional studies reported reduced endothelial apoptosis after subarachnoid hemorrhage and enhanced Nrf2-associated antioxidant responses in diabetic retinal injury [78,79]. Rd reduced retinal vascular leakage while increasing CPT1A-associated fatty acid oxidation and improving redox homeostasis [45]. Rg1, Ro, and Rk1 have also been linked to reduced retinal or endothelial permeability through distinct metabolic, inflammatory, or cytoskeletal mechanisms [42,43,80].

Across Section 4.1, Section 4.2 and Section 4.3, Rb1, Rd, Rg1, and Rg3 show relatively broad experimental coverage. This pattern is consistent at the pathway–family level with the PI3K–Akt, AMPK, and MAPK-associated nodes identified in Figure 1B. However, pathway overlap does not demonstrate equivalent target engagement, potency, or functional synergy among compounds.

**Table 5:** Experimental evidence for ginsenoside-associated regulation of vascular permeability and barrier integrity.

Type	Model	Biological Indicators	Study Type	Administered Dose	Ref.
Rb1	MCAO/R mice	ZO-1, occludin, VE-cadherin, claudin-5 ↑; TNF-α, IFN-γ and p-NF-κB p65 ↓; vascular leakage ↓	In vivo	3 mg/kg	[40]
	LPS-induced rat mesenteric hyperpermeability	p-Cav-1 and p-VE-cadherin ↓; ZO-1 degradation ↓; albumin leakage ↓; NF-κB/Src-associated signaling ↓	In vivo	5 mg/kg	[41]
	Modified double-hemorrhage model	p53, Bax, caspase-3 ↓; Bcl-2 ↑	In vivo	20 mg/kg	[78]
	STZ-induced diabetic Wistar rats	GCLM, GCLC, Nrf2 and GSH ↑; MDA ↓	In vivo	20/40 mg/kg, i.p.	[79]
Rd	Diabetic C57BL/6J mice	CPT1A and FAO ↑; VEGF, IL-6, TNF-α, ROS and MDA ↓; GSH/GSSG ratio ↑; retinal vascular leakage ↓	In vivo	5 μL of 0.5% or 1% (*w*/*v*) Rd eye drops	[45]
Rg1	STZ-induced DR rats; HRMECs	Retinal vascular leakage ↓; GLUT1 ↓; GLUT4 ↑; miR-100-3p ↓; FBXW7 ↑; c-MYC, CD31 and VEGF ↓	In vivo/in vitro	30/60 mg/kg/day, gavage	[42]
	db/db mice	TG and HbA1c ↓; Bcl-2/Bax ratio ↑; caspase-3 ↓	In vivo	25/50 mg/kg	[81]
Ro	SD rats with early diabetic retinopathy	ICAM-1, VCAM-1, IL-1β, TNF-α, IL-6 ↓; Epac1 and p-AMPK ↑	In vivo	10/20 mg/kg/day	[43]
Rk1	HRECs; C57BL/6J mice	VEGF-associated vascular leakage ↓; thrombin- and histamine-induced endothelial permeability ↓	In vitro/in vivo	10 μg/mL in vitro; 1/5/10/15 μg in vivo	[80]

Note: ↑ and ↓ indicate increases and decreases, respectively, relative to the corresponding model/control comparison reported in the cited study. p-, phosphorylated; MCAO/R, middle cerebral artery occlusion/reperfusion; ZO-1, zonula occludens-1; VE-cadherin, vascular endothelial cadherin; TNF-α, tumor necrosis factor alpha; IFN-γ, interferon gamma; NF-κB, nuclear factor kappa B; Cav-1, caveolin-1; GCLM, glutamate-cysteine ligase modifier subunit; GCLC, glutamate-cysteine ligase catalytic subunit; Nrf2, nuclear factor erythroid 2-related factor 2; GSH, reduced glutathione; MDA, malondialdehyde; CPT1A, carnitine palmitoyltransferase 1A; FAO, fatty acid oxidation; VEGF, vascular endothelial growth factor; IL, interleukin; ROS, reactive oxygen species; GSSG, oxidized glutathione; STZ, streptozotocin; DR, diabetic retinopathy; HRMECs, human retinal microvascular endothelial cells; HRECs, human retinal endothelial cells; GLUT1/4, glucose transporter 1/4; miR, microRNA; FBXW7, F-box and WD repeat domain-containing 7; c-MYC, MYC proto-oncogene protein; CD31, cluster of differentiation 31; TG, triglycerides; HbA1c, glycated hemoglobin A1c; Epac1, exchange protein directly activated by cAMP 1; AMPK, AMP-activated protein kinase.

## 5. Ginsenoside-Associated Regulation of Hemostatic and Inflammatory Vascular Responses

### 5.1. Hemodynamic Stress and Platelet Activation

**Figure 4:**
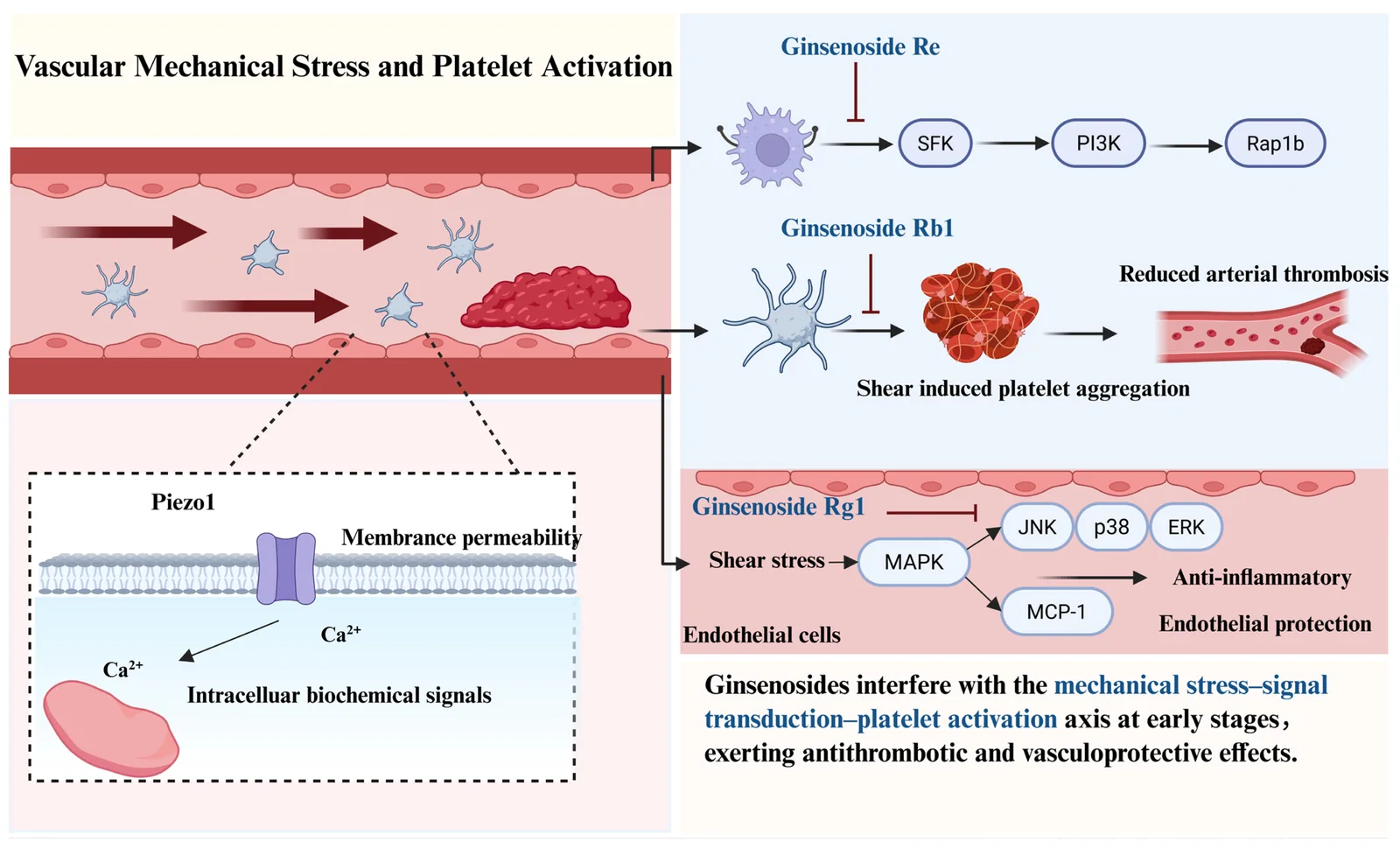
Ginsenoside-associated regulation of hemodynamic stress and platelet activation. SFK, Src family kinase; PI3K, phosphoinositide 3-kinase; Rap1b, Ras-related protein 1b; αIIbβ3, integrin αIIbβ3; ERK, extracellular signal-regulated kinase; MAPK, mitogen-activated protein kinase; JNK, c-Jun N-terminal kinase; p38, p38 mitogen-activated protein kinase; MCP-1, monocyte chemoattractant protein-1; EC, endothelial cell.

As shown in Figure 4, Hemodynamic shear stress influences endothelial and platelet function, whereas disturbed or excessive shear can promote endothelial activation, platelet aggregation, and arterial thrombosis. Although mechanosensitive channels such as Piezo1 participate in vascular mechanotransduction, current evidence does not establish direct interaction between ginsenosides and Piezo1.

Rb1 reduced shear-induced platelet aggregation and arterial thrombosis in experimental models [49], whereas Re inhibited high-shear-induced platelet activation through the SFK/PI3K/Rap1b/αIIbβ3 pathway [46]. Rg1, in contrast, attenuated shear-associated endothelial inflammatory signaling involving ERK, p38, JNK, and MCP-1 [82]. These findings indicate that ginsenosides have been associated with both platelet and endothelial responses to abnormal mechanical stress, although these effects were examined in different experimental systems.

### 5.2. Platelet and Coagulation Responses

Hemostatic balance depends on platelet adhesion and aggregation, coagulation-factor activation, and endogenous anticoagulant mechanisms. Several ginsenosides have been reported to modulate these processes through distinct signaling pathways.

CK reduced collagen-induced platelet adhesion and aggregation [48]. Rk1 inhibited granule release and αIIbβ3 activation, while Rk3 suppressed platelet activation through cAMP/PKA- and PI3K/MAPK-associated signaling [47,83]. Ro also reduced platelet activation through VASP-associated mechanisms [50]. At the coagulation-factor level, Rg1 displayed antithrombin III-dependent anticoagulant activity, whereas Rg2 directly inhibited thrombin and factor Xa in experimental assays [84].

These observations distinguish predominantly platelet-directed effects from coagulation-factor-related actions. However, the available studies do not establish whether these mechanisms operate concurrently in vivo or produce additive antithrombotic effects.

### 5.3. Vascular Inflammation and Oxidative Stress

Oxidative stress and inflammation are closely coupled in vascular disease and contribute to endothelial activation, vascular remodeling, and atherothrombotic progression [57,85,86,87]. The studies summarized in Table 6 identify several recurrent mechanisms, particularly regulation of reactive oxygen species, NF-κB and MAPK signaling, inflammasome activation, and macrophage phenotype.

Rb1 has been associated with reduced inflammatory cytokine production, NF-κB-related signaling, and macrophage polarization in experimental vascular injury and atherosclerosis [51,86,88,89]. Rd and Rb3 have been linked to attenuation of oxidative stress in cerebral ischemic and hypertensive models [34,57,90], whereas Rg3, Rh1, CK, and Rg5 have been associated with suppression of inflammatory or redox signaling involving NLRP3, NF-κB, MAPKs, and antioxidant pathways [52,53,54,55,56].

Nrf2 is a recurrent antioxidant regulator in studies of Rb1, Rg3, and Rh1 [52,53,72,79,91,92]. Because Nrf2 activity can also be influenced by Akt/GSK3β/Fyn-dependent signaling [93,94], its relationship with metabolic and vascular stress is considered further in Section 6.2. Evidence for a complete ginsenoside–GSK3β–Nrf2 signaling sequence, however, remains limited.

At the network level, the experimental findings are broadly consistent with the inflammatory, MAPK, cyclic-nucleotide, and inflammasome-associated nodes identified in Figure 1B. More specifically, the recurrent AMPK- and MAPK-related responses observed experimentally show pathway-level correspondence with PRKAA1, MAPK10, and MAP2K6, which were among the relatively high-degree targets in the network. Together with the PDPK1- and PLCG1-associated relationships discussed in Section 4.2, these observations provide a direct link between the network topology and the mechanistic evidence summarized in Table 3, Table 4, Table 5 and Table 6. Importantly, such concordance is hypothesis-generating and should not be interpreted as evidence of direct target engagement, binding affinity, or causal dependence on these individual proteins. Platelet-related studies have emphasized Re, Rk3, Ro, and CK, whereas Rb1, Rb2, Rd, Rg3, Rh1, CK, and Rg5 have been studied more frequently in inflammatory and oxidative contexts. This distribution indicates overlapping but heterogeneous pathway coverage and does not demonstrate pharmacological additivity, synergy, or coordinated restoration of vascular homeostasis.

**Table 6:** Experimental evidence for ginsenoside-associated regulation of vascular inflammation and oxidative stress.

Type	Model	Biological Indicators	Study Type	Administered Dose	Ref.
Rb1	BALB/c mice with Kawasaki disease-associated coronary artery lesions	TNF-α, IL-6, IL-1β ↓; p-AMPK/AMPK, p-mTOR/mTOR and p-P70S6/P70S6 ↓; p-PI3K/PI3K, p-AKT/AKT and p-GSK3β/GSK3β ↑	In vivo	50/100 mg/kg/day, gavage, 20 days	[89]
	ApoE^−/−^ mice	miR-33 ↓; PEDF ↑; IL-1β, IL-6 and TNF-α ↓; adventitial vasa vasorum and plaque burden ↓	In vivo	50 mg/kg/day, i.p., 4 weeks	[88]
	ApoE^−/−^ mice; mouse peritoneal macrophages	Arg-1, CD206 and p-STAT6 ↑; iNOS and MMP-9 ↓; M2 polarization ↑	In vivo/in vitro	50 mg/kg/day, 7 weeks; 10–80 μM in vitro	[86]
Rb2	HUVECs/THP-1 cells	GRP78, CHOP ↓; p-AMPK and HO-1 ↑; TNF-α, IL-6, IL-1β ↓	In vitro	10–50 μM	[23]
Rb3	Renal artery rings from SHR and WKY rats	NADPH oxidase activity, ROS, p67^phox^, NOX2 and NOX4 ↓	Ex vivo	0.1–1 μM	[34]
Rd	Aged mice with focal cerebral ischemia	CAT, SOD2, GPX and GST ↑; redox imbalance ↓	In vivo	0.1–200 mg/kg, i.p.	[90]
	MCAO/R model	IL-1β, IL-6, IL-18, TNF-α, IFN-γ ↓; p-IκBα and p65 nuclear translocation ↓	In vivo	10 mg/kg, i.p.	[57]
	C57BL/6 mice with cardiac hypertrophy	p-ERK1/2, p-AKT, TGF-β, NOX2, NOX4, ANP and BNP ↓	In vivo	50 μg/kg/day	[95]
Rg3	SD rats with Ang II-induced myocardial hypertrophy	ANP, BNP, β-MHC, collagen I, TGF-β, NLRP3, NF-κB, ROS and MDA ↓; HO-1, Nrf2 and SIRT1 ↑	In vivo	30 mg/kg	[52]
Rh1	Vascular endothelial cells	ROS, MDA and Bax ↓; SOD, Nrf2, HO-1, SOD1 and Bcl-2 ↑	In vitro	25/50 μg/mL	[53]
	HUVECs	TLR2, TLR4, p-STAT3, VCAM-1 and ICAM-1 ↓; NF-κB- and ER-stress-associated signaling ↓	In vitro	25/50 μM	[54]
CK	RAW264.7 macrophages	ABCA1, ABCG1, Atg5, Beclin-1 and LC3-II/LC3-I ↑; p62, IL-1β, iNOS and TNF-α ↓; NF-κB, p38 and JNK signaling ↓	In vitro	2.5 μg/mL	[55]
Rg5	H9c2 cells; C57BL/6 mice	CK-MB, ANP, IL-1β, IL-6, TNF-α, p-JNK/AP-1, β-MyHC, TGF-β1 and collagen I ↓	In vivo/in vitro	0.1–80 μM; 50 mg/kg/day, p.o.	[56]

Note: ↑ and ↓ indicate increases and decreases, respectively, relative to the corresponding model/control comparison reported in the cited study. p-, phosphorylated; TNF-α, tumor necrosis factor alpha; IL, interleukin; AMPK, AMP-activated protein kinase; mTOR, mechanistic target of rapamycin; PI3K, phosphoinositide 3-kinase; Akt, protein kinase B; GSK3β, glycogen synthase kinase 3 beta; miR, microRNA; PEDF, pigment epithelium-derived factor; Arg-1, arginase-1; STAT6, signal transducer and activator of transcription 6; iNOS, inducible nitric oxide synthase; MMP-9, matrix metalloproteinase 9; GRP78, glucose-regulated protein 78; CHOP, C/EBP homologous protein; HO-1, heme oxygenase-1; SHR, spontaneously hypertensive rat; WKY, Wistar–Kyoto rat; NADPH, nicotinamide adenine dinucleotide phosphate; ROS, reactive oxygen species; NOX2/4, NADPH oxidase 2/4; CAT, catalase; SOD, superoxide dismutase; GPX, glutathione peroxidase; GST, glutathione S-transferase; MCAO/R, middle cerebral artery occlusion/reperfusion; IFN-γ, interferon gamma; IκBα, inhibitor of nuclear factor kappa B alpha; ERK, extracellular signal-regulated kinase; TGF-β, transforming growth factor beta; ANP, atrial natriuretic peptide; BNP, B-type natriuretic peptide; β-MHC, beta-myosin heavy chain; NLRP3, NLR family pyrin domain-containing 3; NF-κB, nuclear factor kappa B; MDA, malondialdehyde; Nrf2, nuclear factor erythroid 2-related factor 2; SIRT1, sirtuin 1; TLR2/4, Toll-like receptor 2/4; STAT3, signal transducer and activator of transcription 3; ER, endoplasmic reticulum; ABCA1, ATP-binding cassette transporter A1; ABCG1, ATP-binding cassette transporter G1; Atg5, autophagy-related 5; LC3, microtubule-associated protein 1 light chain 3; JNK, c-Jun N-terminal kinase; AP-1, activator protein 1; CK-MB, creatine kinase-MB; HUVECs, human umbilical vein endothelial cells; THP-1, human monocytic leukemia cell line; VECs, vascular endothelial cells.

## 6. Insulin Resistance, GSK3β–Nrf2 Crosstalk, and Neurovascular Implications

### 6.1. Insulin Resistance and Vascular Dysfunction

Insulin resistance is relevant to vascular homeostasis because impaired insulin signaling affects not only systemic glucose metabolism but also endothelial signaling, vasomotor regulation, inflammatory activation, and vascular remodeling. At the cerebral level, impaired insulin signaling is likewise pertinent to the neurovascular interface, where endothelial function, blood–brain barrier integrity, cerebral perfusion, and neurovascular-unit homeostasis are closely interconnected. This vascular perspective provides the rationale for considering brain insulin resistance and the conceptual “type 3 diabetes” framework later in Section 6.3, rather than treating them solely as manifestations of neuronal pathology [11,12].

Insulin resistance is a central feature of metabolic syndrome and type 2 diabetes mellitus and contributes to both macrovascular and microvascular complications. In endothelial cells, insulin activates PI3K/Akt/eNOS signaling and promotes nitric oxide production, whereas impaired endothelial insulin signaling reduces vasodilation, enhances inflammatory activation and leukocyte adhesion, and accelerates atherosclerosis [96,97]. Hyperglycemia, dyslipidemia, oxidative stress, and chronic inflammation further aggravate endothelial dysfunction and vascular remodeling [22,25,85,98].

Several pathways modulated by ginsenosides overlap with those disturbed during insulin resistance. The bioinformatics analysis identified insulin signaling, insulin resistance, adipocytokine signaling, PI3K–Akt signaling, and lipid and atherosclerosis among the enriched pathways (Figure 1). Experimentally, Rb1 has been associated with SIRT1/AMPK- and SIRT1/eNOS-related endothelial protection [21,33], while Rg3, Rg1, Rd, Rg2, and Ro have been linked to PI3K/Akt, PPARγ, oxidative stress, inflammatory signaling, and vascular remodeling in diabetic or ischemic settings [29,30,31,32,42,43,44,52,60,68,81,99].

These findings indicate mechanistic overlap but do not establish that ginsenosides directly reverse vascular insulin resistance. Most studies assessed individual pathways or vascular outcomes without simultaneously measuring systemic insulin sensitivity, endothelial insulin signaling, and vascular function. Accordingly, the relevance of insulin resistance to this review lies in its capacity to disrupt both peripheral vascular homeostasis and, potentially, the cerebral neurovascular interface, thereby providing a mechanistic bridge between cardiometabolic vascular injury and the brain insulin-resistance context discussed below. Studies integrating metabolic phenotyping, endothelial insulin receptor/IRS/PI3K/Akt signaling, vascular reactivity, blood–brain barrier or neurovascular-unit endpoints where appropriate, and pharmacokinetic exposure are therefore needed.

### 6.2. GSK3β–Nrf2 Crosstalk in Metabolic and Vascular Stress

Glycogen synthase kinase-3β (GSK3β) and nuclear factor erythroid 2-related factor 2 (Nrf2) provide a potential link between impaired insulin signaling and vascular oxidative stress. GSK3β is regulated downstream of PI3K/Akt, whereas Nrf2 controls antioxidant and cytoprotective gene expression. In addition to canonical Keap1-dependent regulation, GSK3β can promote Fyn-dependent nuclear export and degradation of Nrf2; inhibition of this pathway favors nuclear Nrf2 accumulation and antioxidant transcription [93]. In diabetic endothelial progenitor cells, disruption of Akt/GSK3β/Fyn signaling was associated with reduced Nrf2 activity and impaired antioxidant and angiogenic responses, supporting the vascular relevance of this pathway [94].

Among ginsenosides, evidence is stronger for Nrf2 modulation than for direct regulation of the complete GSK3β–Nrf2 axis. Rb1 promoted Nrf2-dependent vascular protection and reduced inflammatory adhesion in endothelial and atherosclerotic models [91,92]. Mechanistic studies further linked Rb1 to Keap1 degradation, Nrf2 nuclear translocation, and suppression of NOX2-associated oxidative stress [92]. Rg3 and Rh1 have likewise been associated with Nrf2/HO-1 activation in myocardial, cerebral ischemic, and endothelial injury models [52,53,72].

Rb1 has also been reported to alter PI3K/Akt/GSK3β-associated signaling in Kawasaki disease-associated coronary artery lesions [89]. However, because the GSK3β phosphorylation site was not specified and Nrf2/Fyn signaling was not assessed in the same experiment, these findings cannot establish GSK3β inhibition or a sequential Rb1–Akt/GSK3β–Fyn–Nrf2 mechanism. Thus, GSK3β–Nrf2 crosstalk remains a plausible mechanistic link between insulin resistance and the vascular actions of ginsenosides rather than a demonstrated common pathway.

Future studies should assess site-specific GSK3β phosphorylation, Fyn localization, Nrf2 nuclear translocation, downstream antioxidant genes, oxidative stress, and vascular function within the same exposure-matched experimental system.

### 6.3. Brain Insulin Resistance, Amyloid/Tau Pathology, and the Neurovascular Interface

Impaired cerebral insulin and insulin-like growth factor signaling has been described in Alzheimer’s disease (AD), giving rise to the conceptual term “type 3 diabetes” for brain insulin resistance associated with neurodegenerative abnormalities [11,12]. Insulin resistance, oxidative stress, vascular dysfunction, amyloid-β (Aβ) accumulation, and tau hyperphosphorylation may interact in AD, with GSK3β providing a potential link between impaired metabolic signaling and tau pathology.

Experimental studies indicate that selected ginsenosides influence Aβ-associated processes. In an Aβ1–42-induced rat model, Rg1 reduced hippocampal Aβ1–42 and increased PPARγ and insulin-degrading enzyme expression [100]. In 5XFAD mice, Rg1 also reduced Aβ deposition and improved PINK1/Parkin-dependent mitophagy [101]. These findings connect ginsenoside activity with amyloid metabolism and mitochondrial quality control, but do not demonstrate correction of brain insulin resistance.

Ginsenosides have also been examined in relation to tau phosphorylation. Rg1 attenuated GSK3β/tau-associated abnormalities and Aβ formation in an okadaic acid-induced AD-like model [102]. In APP transgenic mice, Rd reduced tau phosphorylation at Ser199/202, Ser396, and Ser404, accompanied by changes in GSK3β Tyr216 phosphorylation and CDK5/P25/P35 signaling [103]. These observations support modulation of tau-related pathways but remain preclinical.

From the perspective of vascular homeostasis, the neurodegenerative evidence should be interpreted cautiously. Most studies did not simultaneously assess cerebral endothelial insulin signaling, blood–brain barrier integrity, cerebral perfusion, or vascular target engagement. Accordingly, reductions in Aβ or phosphorylated tau cannot be attributed to restoration of cerebral vascular homeostasis, nor do these findings establish efficacy against AD in humans. Future studies should integrate cerebral metabolic phenotyping, Aβ and site-specific tau phosphorylation, neurovascular-unit and blood–brain barrier measurements, pathway perturbation, and pharmacokinetic assessment.

## 7. Context-Dependent Regulation of Angiogenesis by Ginsenosides

**Figure 5:**
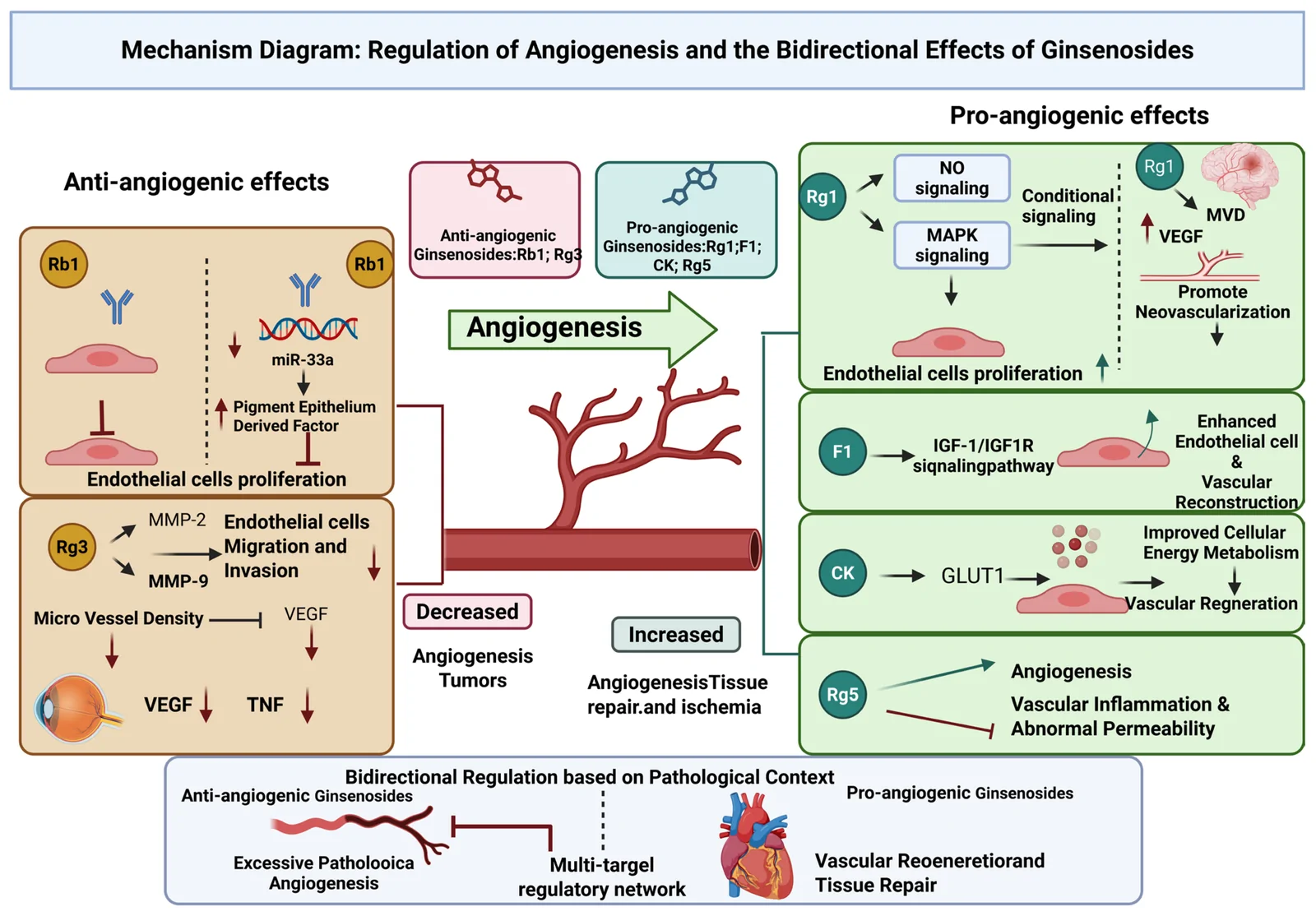
Schematic overview of the context-dependent pro- and antiangiogenic effects of representative ginsenosides. MVD, microvessel density; VEGF, vascular endothelial growth factor; TNF-α, tumor necrosis factor alpha; PEDF, pigment epithelium-derived factor; MMP, matrix metalloproteinase; PI3K, phosphoinositide 3-kinase; Akt, protein kinase B; mTOR, mechanistic target of rapamycin; SIRT3, sirtuin 3; IGF-1, insulin-like growth factor 1; IGF-1R, insulin-like growth factor 1 receptor; HIF-1α, hypoxia-inducible factor 1 alpha; GLUT1, glucose transporter 1; ERK, extracellular signal-regulated kinase; eNOS, endothelial nitric oxide synthase; CK, compound K.

Angiogenesis contributes to tissue repair and post-ischemic recovery but also to tumor progression and diabetic microvascular disease [104]. Experimental evidence indicates that the angiogenic effects of ginsenosides depend on the compound and pathological context rather than following a uniform pro- or antiangiogenic pattern (Figure 5 and Table 7).

Rb1 and Rg3 have been studied primarily in settings of pathological angiogenesis. Rb1 inhibited endothelial tube formation through PEDF-associated mechanisms [62,105], whereas Rg3 reduced endothelial migration and angiogenic activity through suppression of MMP-2/MMP-9 and VEGF-related signaling [58,59,106,107,108]. These effects were observed mainly in tumor, endometriosis, and diabetic retinal models and therefore should not be generalized to physiological or reparative angiogenesis.

In contrast, F1, CK, and Rg5 have been associated mainly with angiogenic responses in ischemic or regenerative settings. F1 promoted post-ischemic vascular reconstruction through IGF-1/IGF1R signaling [61], whereas CK enhanced endothelial angiogenic activity and vascular repair in fracture and cerebral ischemia models [63,109]. Rg5 also promoted angiogenesis and perfusion recovery after hindlimb ischemia through IGF-1R/ERK/Akt/eNOS-associated signaling [39].

Rg1 illustrates the strongest context dependence. In high-glucose-treated retinal endothelial cells, Rg1 suppressed VEGF expression, endothelial proliferation, migration, and tube formation through the SNHG7/miR-2116-5p/SIRT3 axis [99]. By contrast, in cerebral ischemia, Rg1 increased VEGF-associated signaling and post-ischemic angiogenesis through PI3K/Akt/mTOR-related mechanisms [60,110]. Thus, the direction of the angiogenic response cannot be assigned solely on the basis of ginsenoside identity.

At the network level, angiogenesis illustrates both pathway overlap and functional divergence among ginsenosides. PI3K–Akt, MAPK, IGF-related, VEGF-associated, and extracellular-matrix pathways recur across different compounds, but the biological outcome varies with vascular bed and disease context. The available evidence therefore supports context-dependent regulation of angiogenesis rather than a general pro- or antiangiogenic property of ginsenosides and does not establish therapeutic complementarity or synergy.

**Table 7:** Experimental evidence for context-dependent effects of ginsenosides on angiogenesis.

Type	Model	Biological Indicators	Study Type	Administered Dose	Ref.
Rb1	HUVECs	miR-33a ↓; PEDF ↑; endothelial tube formation ↓	In vitro	0–20 nM	[62]
Rg3	HUVECs	MMP-2, MMP-9 ↓; migration/invasion-associated angiogenic responses ↓	In vitro	1–1000 nM	[58]
	BALB/c mice with breast tumors	MVD and VEGF ↓	In vivo	10 mg/kg/day, p.o.	[106]
	SD rats with endometriosis	VEGF, p-Akt and p-mTOR ↓	In vivo	3/10 mg/kg/day, p.o.	[59]
	C57BL/6 mice with lung tumors	VEGF and MVD ↓	In vivo	20 mg/kg/day, i.p.	[107]
	Wistar diabetic rats	Retinal VEGF and TNF-α ↓	In vivo	5 mg/kg	[108]
Rg1	High-glucose-treated HRECs	lncRNA SNHG7 ↑; miR-2116-5p ↓; SIRT3 ↑; VEGF ↓; proliferation, migration and tube formation ↓	In vitro	10 μM	[99]
	C57BL/6 mice with cerebral ischemia; hCMEC/D3 cells	BrdU^+^/CD31^+^ cells ↑; p-Akt, p-mTOR, HIF-1α and VEGF ↑; post-ischemic angiogenesis ↑	In vivo/in vitro	10/20/40 mg/kg/day; 0.1–1000 μM	[60]
F1	MCAO rats	Microvascular density and focal cerebral blood perfusion ↑; post-ischemic angiogenesis ↑	In vivo	50 mg/kg/day, 14 days	[61]
CK	BMSC–HUVEC co-culture	Endothelial tube length and branch points ↑; angiogenic activity ↑	In vitro	10 μM	[109]
	SD rats with open femoral fracture	CD31^high expression^Emcn^high expression^ H-type vessels ↑; fracture-associated angiogenesis ↑	In vivo	500 μM, 100 μL local injection every other day for 4 weeks	[109]
	MCAO/R SD rats; BMSC-derived brain microvascular endothelial-like cells	FLK-2, CD31, VEGF, HIF-1α, GLUT1 and ATP ↑; angiogenesis ↑	In vivo/in vitro	28 mg/kg/day, intragastric, 30 days; 10 μM in vitro	[63]
Rg5	C57BL/6J mice with hindlimb ischemia	p-ERK, p-Akt, p-Src and eNOS-associated signaling ↑; blood-flow recovery and capillary formation ↑	In vivo	300 μmol/100 μL per mouse, intramuscular administration	[39]

Note: ↑ and ↓ indicate increases and decreases, respectively, relative to the corresponding experimental comparison reported in the cited study. p-, phosphorylated; HUVECs, human umbilical vein endothelial cells; HRECs, human retinal endothelial cells; MVD, microvessel density; VEGF, vascular endothelial growth factor; PEDF, pigment epithelium-derived factor; MMP, matrix metalloproteinase; TNF-α, tumor necrosis factor alpha; lncRNA, long non-coding RNA; miR, microRNA; SNHG7, small nucleolar RNA host gene 7; SIRT3, sirtuin 3; BrdU, 5-bromo-2′-deoxyuridine; CD31, cluster of differentiation 31; hCMEC/D3, human cerebral microvascular endothelial cell line D3; HIF-1α, hypoxia-inducible factor 1 alpha; PI3K, phosphoinositide 3-kinase; Akt, protein kinase B; mTOR, mechanistic target of rapamycin; BMSCs, bone marrow-derived mesenchymal stem cells; IGF-1, insulin-like growth factor 1; IGF-1R, insulin-like growth factor 1 receptor; Emcn, endomucin; CD31^hi Emcn^hi, endothelial cells with high CD31 and endomucin expression; MCAO/R, middle cerebral artery occlusion/reperfusion; GLUT1, glucose transporter 1; ATP, adenosine triphosphate; ERK, extracellular signal-regulated kinase; eNOS, endothelial nitric oxide synthase.

## 8. Summary and Perspectives

The available evidence indicates that individual ginsenosides modulate multiple components of vascular homeostasis, including endothelial function, vascular remodeling and tone, barrier integrity, hemostatic responses, inflammation, oxidative stress, and angiogenesis. These effects involve partially overlapping signaling pathways, but most evidence derives from isolated compounds studied in heterogeneous cell and animal models. The available data therefore support regulation of selected vascular processes rather than coordinated restoration of vascular homeostasis. Direct comparisons among ginsenosides and experimental demonstration of additivity or synergy remain limited.

Structural variation and biotransformation are likely to contribute to differences in the pharmacological and pharmacokinetic profiles of ginsenosides. This issue is particularly relevant because several in vitro studies employed concentrations substantially exceeding plasma levels reported after oral ginseng administration. Future studies should therefore compare individual compounds and defined combinations under standardized conditions and use exposure ranges informed by human pharmacokinetic data. Integration of circulating metabolites, tissue distribution, target engagement, and vascular functional endpoints will be necessary to determine whether experimentally identified mechanisms operate at physiologically achievable exposures. Microbial biotransformation should also be considered because it can substantially alter the systemic availability of less glycosylated ginsenosides and metabolites.

Insulin resistance provides an additional metabolic context in which impaired endothelial signaling, inflammation, oxidative stress, and vascular dysfunction converge [96,97]. Several pathways affected by ginsenosides, including AMPK, PI3K/Akt, eNOS, PPARγ, and NF-κB, overlap with mechanisms disrupted during cardiometabolic insulin resistance [21,30,32,33,52,68]. Brain insulin resistance and the proposed concept of “type 3 diabetes” further extend this relationship to the neurovascular abnormalities associated with Alzheimer’s disease [11,12]. Experimental studies have reported effects of selected ginsenosides on Aβ-associated pathology, tau hyperphosphorylation, and mitochondrial quality control [100,101,102,103]. However, these findings remain predominantly preclinical and do not establish that ginsenosides correct systemic or cerebral insulin resistance or that changes in Aβ or phosphorylated tau result from restoration of neurovascular function.

The GSK3β–Nrf2 relationship represents a potential mechanistic link between impaired insulin signaling and oxidative vascular injury. Experimental evidence supports GSK3β/Fyn-dependent regulation of Nrf2 [93,94], while Rb1, Rg3, and Rh1 have been associated with Nrf2-related antioxidant responses in several vascular or vascular-related models [52,53,72,79,91,92]. Rb1 has also been linked separately to PI3K/Akt/GSK3β-associated signaling [89]. However, a sequential ginsenoside–Akt/GSK3β–Fyn–Nrf2 mechanism has not been demonstrated within the same vascular experimental system. Site-specific GSK3β phosphorylation, Nrf2 nuclear translocation, pathway dependence, and vascular functional outcomes should therefore be assessed together in future studies.

The compound–target–pathway analysis presented in this review identifies shared and compound-specific signaling patterns but remains predictive and requires experimental validation. Further progress will depend on comparative and exposure-matched studies, direct testing of compound interactions and pathway dependence, and clinical evaluation of pharmacokinetic and vascular outcomes. These approaches are required to determine whether the multi-target actions observed experimentally can be translated into clinically relevant vascular effects.

## Figures and Tables

**Table 1:** Representative ginsenosides classified according to aglycone type and glycosylation pattern.

Type	Ginsenosides	R1	R2
PPD	Rb1	-glc(2-1) glc	-glc (6-1) glc
	Rb2	-glc (2-1) glc	-glc (6-1) ara (p)
	Rb3	-glc (2-1) glc	-glc (6-1) xyl
	Rc	-glc (2-1) glc	-glc(6-1) ara (f)
	Rd	-glc (2-1) glc	-glc
	Rg3	-glc (2-1) glc	-H
	Rh2	-glc	-H
	F2	-glc	-glc
PPT	Re	-glc(2-1)glc	-glc
	Rf	-glc (2-1) glc	-H
	Rg1	-glc	-glc
	Rg2	-glc (2-1) rha	-H
	Rh1	-glc	-H
	F1	-H	-glc
Oleanolic acid	Ro	-glcUA (2-1) glc	-glc

**Table 2:** Mechanistic pathways underlying the vascular effects of ginsenosides.

Vascular-Homeostasis Domain	Representative Ginsenosides	Recurrent Mechanisms/Pathways	Representative Refs.
Vascular structure and remodeling	Rb1, Rd, Rg3, Rg2	SIRT1/AMPK, autophagy, PI3K/Akt, PPARγ/FAK, TGF-β/Smad	[21,28,29,30,31,32]
Vascular tone	Rb1, Rb3, Rd, Rg1, Rg5	eNOS/NO, Ca^2+^ handling, NADPH oxidase, MAPK, IGF-1R/Akt/eNOS	[33,34,35,36,37,38,39]
Barrier integrity	Rb1, Rd, Rg1, Ro	PPARγ, junctional proteins, AMPK, redox regulation	[40,41,42,43,44,45]
Platelet and hemostatic responses	Rb1, Re, Rk3, Ro, CK	SFK/PI3K/Rap1b, cAMP/PKA, αIIbβ3, platelet adhesion	[46,47,48,49,50]
Inflammation and oxidative stress	Rb1, Rb2, Rb3, Rd, Rg3, Rh1, CK, Rg5	NF-κB, MAPK, Nrf2/HO-1, NADPH oxidase, NLRP3	[23,34,51,52,53,54,55,56,57]
Angiogenesis	Rb1, Rg3, Rg1, F1, CK, Rg5	PPARγ/PEDF, VEGF, PI3K/Akt/mTOR, IGF-1/IGF1R	[39,58,59,60,61,62,63]

Note: The listed pathways summarize representative experimental findings and do not imply that all mechanisms have been demonstrated for each compound or within the same experimental model. SIRT1, sirtuin 1; AMPK, AMP-activated protein kinase; PI3K, phosphoinositide 3-kinase; Akt, protein kinase B; PPARγ, peroxisome proliferator-activated receptor gamma; FAK, focal adhesion kinase; TGF-β, transforming growth factor beta; Smad, Smad family signaling protein; eNOS, endothelial nitric oxide synthase; NO, nitric oxide; NADPH, nicotinamide adenine dinucleotide phosphate; MAPK, mitogen-activated protein kinase; IGF-1R, insulin-like growth factor 1 receptor; SFK, Src family kinase; Rap1b, Ras-related protein 1b; cAMP, cyclic adenosine monophosphate; PKA, protein kinase A; αIIbβ3, integrin αIIbβ3; NF-κB, nuclear factor kappa B; Nrf2, nuclear factor erythroid 2-related factor 2; HO-1, heme oxygenase-1; NLRP3, NLR family pyrin domain-containing 3; PEDF, pigment epithelium-derived factor; VEGF, vascular endothelial growth factor; mTOR, mechanistic target of rapamycin; IGF-1, insulin-like growth factor 1.

**Table 3:** Experimental evidence for ginsenoside-associated maintenance of vascular structure and remodeling.

Type	Model	Biological Indicators	Study Type	Administered Dose	Ref.
Rb1	HUVECs	PAI-1 ↓; eNOS and NO ↑; SIRT1 and p-AMPK ↑; ATP ↑	In vitro	10/20 μM	[21]
	Aged C57BL/6J mice	p21^Cip1^/p16^INK4a^ ↓; collagen I and III ↓; ICAM-1, VCAM-1, PAI-1 ↓	In vivo	10/20 mg/kg/day, i.p.	[65]
	HUVECs/PBMCs/VSMCs	CCND1, CDK4 ↓	In vitro	80 μM (HUVECs), 60 μM (PBMCs), and 320 μM (VSMCs)	[24]
	MCAO/R model	Bax and caspase-3 ↓; Bcl-2 ↑; p-PERK, ATF6, GRP78, CHOP, p-IRE1, p-JNK ↓	In vivo	20/40 mg/kg/day	[66]
	ApoE^−/−^ mice	Atherosclerotic plaque area and inflammatory cytokines ↓; cleaved caspase-3/9 ↓; LC3-II/LC3-I and BECN1 ↑; p62 ↓	In vivo	10 mg/kg/day, intragastric, 8 weeks	[67]
	HFD-fed SD rats; ox-LDL-treated HUVECs	Endothelial senescence/SA-β-gal-positive cells ↓; PAI-1 and p62 ↓; SIRT1 and LC3-II/LC3-I ↑; Beclin-1 acetylation ↓	In vivo/in vitro	40 mg/kg, i.p.; 40 μM	[28]
Rd	MCAO/R model	p-Akt, p-ERK, VEGF, BDNF ↑	In vivo	1/2.5/5 mg/kg/day, i.p.	[29]
Rg3	ApoE^−/−^ mice; HUVECs	VCAM-1, ICAM-1, MCP-1, IL-6 ↓	In vivo/in vitro	15/30 mg/kg/day; 7.5/15/30 μM	[32]
	Diabetic ApoE^−/−^ mice; VSMCs	PPARγ ↑; cyclin D1, MMP-2 and MMP-9 ↓; VSMC proliferation/migration ↓; plaque area ↓	In vivo/in vitro	10 mg/kg, i.p., every 2 days; 10–50 μM	[30]
Rg2	Wistar rats	TGF-β1, p-Smad3, collagen I, α-SMA ↓	In vivo	5/20 mg/kg, p.o.	[31]
	SD rats; VSMCs/HUVECs	TNF-α, IL-6, IL-8, IL-1β, p-ERK1/2 ↓	In vivo/in vitro	8/40 mg/kg/day; 10–40 μM	[68]

Note: ↑ and ↓ indicate increases and decreases, respectively, relative to the corresponding model/control comparison reported in the cited study. p-, phosphorylated; PAI-1, plasminogen activator inhibitor-1; eNOS, endothelial nitric oxide synthase; NO, nitric oxide; SIRT1, sirtuin 1; AMPK, AMP-activated protein kinase; ATP, adenosine triphosphate; p21^Cip1, cyclin-dependent kinase inhibitor 1A; p16^INK4a, cyclin-dependent kinase inhibitor 2A; ICAM-1, intercellular adhesion molecule 1; VCAM-1, vascular cell adhesion molecule 1; CCND1, cyclin D1; CDK4, cyclin-dependent kinase 4; Bax, Bcl-2-associated X protein; Bcl-2, B-cell lymphoma 2; PERK, protein kinase R-like endoplasmic reticulum kinase; ATF6, activating transcription factor 6; GRP78, glucose-regulated protein 78; CHOP, C/EBP homologous protein; IRE1, inositol-requiring enzyme 1; JNK, c-Jun N-terminal kinase; LC3, microtubule-associated protein 1 light chain 3; BECN1, beclin 1; SA-β-gal, senescence-associated β-galactosidase; HFD, high-fat diet; ox-LDL, oxidized low-density lipoprotein; Akt, protein kinase B; ERK, extracellular signal-regulated kinase; VEGF, vascular endothelial growth factor; BDNF, brain-derived neurotrophic factor; MCP-1, monocyte chemoattractant protein-1; IL, interleukin; PPARγ, peroxisome proliferator-activated receptor gamma; MMP, matrix metalloproteinase; TGF-β, transforming growth factor beta; α-SMA, alpha-smooth muscle actin; TNF-α, tumor necrosis factor alpha; HUVECs, human umbilical vein endothelial cells; PBMCs, peripheral blood mononuclear cells; VSMCs, vascular smooth muscle cells; MCAO/R, middle cerebral artery occlusion/reperfusion; i.p., intraperitoneal; p.o., oral administration.

## Data Availability

No new data were created or analyzed in this study. Data sharing is not applicable to this article.

## Supplementary Materials

The following supporting information can be downloaded at: https://www.mdpi.com/article/10.3390/nu18162729/s1, Table S1: Compound-specific target prediction records for the 13 representative ginsenosides; Table S2: Vascular-homeostasis-related genes retrieved from GeneCards and OMIM; Table S3: Intersecting targets between ginsenoside-associated targets and vascular-homeostasis-related genes; Table S4: Complete KEGG pathway enrichment results for the intersecting ginsenoside–vascular-homeostasis target set. The literature sources cited in the Supplementary Materials are included in the main reference list [21,39,47,60,61,111,112,113,114,115,116,117,118,119,120,121,122,123,124,125,126].
